# Reduced Facilitation of Parietal-Motor Functional Connections in Older Adults

**DOI:** 10.3389/fnagi.2021.595288

**Published:** 2021-02-01

**Authors:** Elana R. Goldenkoff, Rachel N. Logue, Susan H. Brown, Michael Vesia

**Affiliations:** ^1^Brain Behavior Laboratory, School of Kinesiology, University of Michigan, Ann Arbor, MI, United States; ^2^Motor Control Laboratory, School of Kinesiology, University of Michigan, Ann Arbor, MI, United States

**Keywords:** aging, posterior parietal cortex (PPC), primary motor cortex (M1), motor evoked potential, transcrancial magnetic stimulation

## Abstract

Age-related changes in cortico-cortical connectivity in the human motor network in older adults are associated with declines in hand dexterity. Posterior parietal cortex (PPC) is strongly interconnected with motor areas and plays a critical role in many aspects of motor planning. Functional connectivity measures derived from dual-site transcranial magnetic stimulation (dsTMS) studies have found facilitatory inputs from PPC to ipsilateral primary motor cortex (M1) in younger adults. In this study, we investigated whether facilitatory inputs from PPC to M1 are altered by age. We used dsTMS in a conditioning-test paradigm to characterize patterns of functional connectivity between the left PPC and ipsilateral M1 and a standard pegboard test to assess skilled hand motor function in 13 young and 13 older adults. We found a PPC-M1 facilitation in young adults but not older adults. Older adults also showed a decline in motor performance compared to young adults. We conclude that the reduced PPC-M1 facilitation in older adults may be an early marker of age-related decline in the neural control of movement.

## Introduction

Age-related decline in cognitive and sensorimotor functions in older adults has been linked with changes in the brain's structural and functional connectivity patterns (Seidler et al., 2010; Damoiseaux, 2017). These age-related differences in functional connectivity that mediate information flow across the brain have been attributed in part to the decline in white matter integrity in older adults (Wu and Hallett, 2005; Zahr et al., 2009; Sullivan et al., 2010; Bruijn, 2014). Additionally, mounting evidence from neuroimaging suggests age-related changes in cortico-cortical connectivity in the motor network of healthy older adults contribute to age-related declines in sensorimotor functions. Functional cortico-cortical connectivity measures derived from dual-site transcranial magnetic stimulation (dsTMS) in healthy older adults also have shown reduced facilitatory and inhibitory inputs from secondary motor areas, including the supplementary motor area (SMA) (Green et al., 2018) and dorsal premotor cortex (PMd) (Ni et al., 2014), to primary motor cortex (M1). Posterior parietal cortex (PPC), a region involved in transforming sensory information into motor commands (Crawford et al., 2003, 2004; Andersen and Cui, 2009), is strongly interconnected with motor areas through white-matter tracts of the superior longitudinal fasciculus (Makris, 2004). These reciprocal glutamatergic parietal-frontal circuits are likely excitatory (Tokuno and Nambu, 2000; Dum and Strick, 2002; Matsumoto et al., 2006) and underlie control processes for skilled voluntary movements such as dexterous finger movements required during the manipulation of objects (Filimon, 2010; Davare et al., 2011; Vesia and Crawford, 2012; Turella and Lingnau, 2014; Gallivan and Culham, 2015).

Human neuroimaging studies have implicated parieto-frontal brain regions in sensorimotor control of human hand behavior (Gallivan et al., 2011, 2013; Fabbri et al., 2014; Monaco et al., 2020; Turella et al., 2020). Anatomical findings in non-human primates have shown direct monosynaptic inputs to M1 from PPC in the control of hand movements (Strick and Kim, 1978; Rozzi et al., 2005; Bruni, 2018). A similar direct functional and anatomical parieto-motor pathway has been seen in human imaging (Koch et al., 2010). A number of dsTMS findings also have shown direct facilitatory parieto-motor connectivity in both the resting and active brain for hand actions in young adults (Koch et al., 2007, 2008; Ziluk et al., 2010; Cattaneo and Barchiesi, 2011; Karabanov et al., 2013; Vesia et al., 2013, 2017). Similarly, recent findings from intraoperative dual cortical stimulation in humans have provided direct evidence that the inferior parietal lobule exerts short-latency excitatory effects on cortical motor output (Cattaneo et al., 2020). Importantly, a recent neuroimaging study points to reduced coupling of parietal and premotor areas as a possible mechanism for the decreased perceptual motor speed observed in older adults (Michely et al., 2018). A question that remains, however, is whether the well-established age-related decline in sensorimotor performance relates to age-related differences in parieto-motor connectivity in older adults. We used dsTMS to characterize patterns of functional PPC-M1 connectivity and a standard pegboard test to estimate skilled motor performance in young and older adults. We hypothesized that facilitatory connectivity between PPC and M1 is reduced in older adults.

## Methods

### Participants

Thirteen young adults (YA, 8 females, 19.9 ± 1.3 years) and thirteen older adults (OA, 5 females, 72.2 ± 5.5 years) provided written consent to participate in the study. All participants were right-handed as assessed by the Edinburgh Handedness Inventory (Oldfield, 1971). All participants were screened for any contraindications to TMS (Keel et al., 2001; Rossi et al., 2011) and had no history of neurological disorders. To assess weekly frequency and duration of various physical activities undertaken by older adults, we administered the Community Health Activities Model Program for Seniors self-report questionnaire (CHAMPS), which revealed that all were very physically active (total caloric expenditure per week: 3,799.5 ± 869.6; (Stewart et al., 2001). Cognitive function was assessed in the older adults using the Montreal Cognitive Assessment (MoCA score, ≥26) (Nasreddine et al., 2005) and Mini-Mental State Exam (MMSE score ≥27) (Folstein et al., 1975). Those who took CNS-active medications within 48 h of the study were excluded. All procedures were approved by the University of Michigan Institutional Review Board (HUM00155459) in accordance with the Declaration of Helsinki.

### Procedures

**Figure 1:**
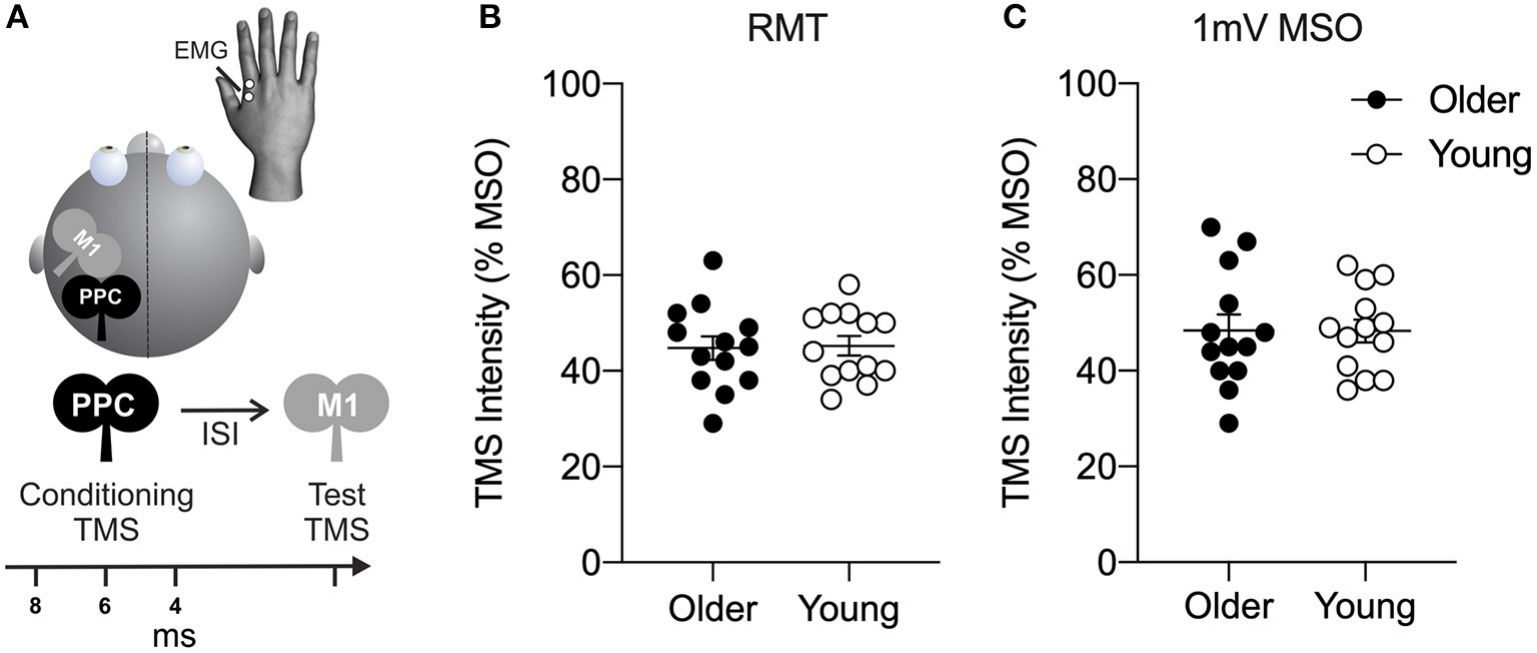
**(A)** Dual-site transcranial magnetic stimulation in a conditioning-test approach was used to probe connectivity between left posterior parietal cortex (PPC) and ipsilateral primary motor cortex (M1). The conditioning stimulus (CS) was delivered at an intensity of 70, 90, 110, or 130% of resting motor threshold (RMT) to the PPC. CS was delivered 4, 6, or 8 ms prior to a test stimulus (TS) delivered to primary motor cortex (M1). Resulting motor evoked potential (MEP) amplitudes were recorded using electromyography (EMG) in the right-hand target muscle at rest. **(B)** Motor excitability plots showing transcranial magnetic stimulation (TMS) intensity (expressed as a percentage of the maximum stimulator output, MSO) of resting motor threshold (RMT) and **(C)** TMS intensity to elicit a motor evoked potential (MEP) of 1 mV for older (filled circles) and young (open circles) adults. Mean and SE are presented.

Transcranial magnetic stimulation in a conditioning-test approach with two coils (Lafleur et al., 2016; Hallett et al., 2017; Goldenkoff et al., 2020) was used to measure connectivity between left PPC and left M1 (Figure 1A). A test stimulus (TS) was delivered to M1 with a figure-8 coil (D70^2^, 7 cm diameter) connected to a Magstim 200^2^ stimulator (Magstim, Whitland, UK) with a monophasic waveform. The TS coil was held tangential to the skull at 45° from the mid-sagittal line, inducing a current in the posterior-anterior direction in the underlying cortical tissue. TS intensity was set to produce a motor evoked potential (MEP) of ~1 mV in the first dorsal interosseous muscle or the abductor pollicis brevis muscle in the right hand (Rossini et al., 2015). Left M1 was defined as the optimal scalp position for coil placement where stimulation evoked the largest MEP from the quiescent right-hand target muscle. PPC stimulation was applied to the P3 electrode position of the international 10-20 electroencephalogram (EEG) coordinate system using commercially available EEG head caps in each participant. The site is situated over angular gyrus (BA 39) of the inferior parietal lobule (Herwig et al., 2004; Okamoto et al., 2004) and corresponds with activation foci for hand actions identified by neuroimaging (Vesia and Crawford, 2012). A conditioning stimulus (CS) was delivered to PPC with another figure-8 coil (D50 Alpha B.I., 5 cm diameter) connected to a Magstim 200^2^ stimulator (Magstim, Whitland, UK) with a monophasic waveform and a posterior-anterior current direction. The CS coil was held tangential to the skull at 90° from the mid-sagittal line. CS preceded TS by an inter-stimulus interval (ISI) of 4, 6, or 8 ms. PPC stimulation intensity was applied at 70, 90, 110, and 130% of resting motor threshold (RMT), similar to previous work (Koch et al., 2007). RMT was defined as the lowest intensity that evoked MEPs of at least 50 μV in peak-to-peak amplitude in three of five consecutive trials with the PPC coil from the right-hand muscle (Rossini et al., 1994). Each stimulus-response curve was repeated for each ISI. Twelve single-pulse stimuli (TS alone) to M1 and paired-pulse stimuli (CS-TS) at each PPC stimulation intensity were delivered in random order within an experimental block (60 trials) with both hands at rest. Stimuli were applied every 5 s. The order of the ISI block for each stimulus-response curve was counterbalanced across participants. A frameless stereotactic neuronavigation system (Brainsight; Rogue Research, Montreal, Canada) was used to ensure consistency in the TMS coil position throughout the stimulation session. After the electrophysiological measurements were completed, motor skill performance was examined by a test of hand dexterity, the Grooved Pegboard Test (GPT, Lafayette Instrument # 32025) using standard procedures (Wang et al., 2011).

### Data Analysis

Electromyography (EMG) signals were recorded from the right-hand target muscle using bipolar surface electrodes (Model 2024F, Intronix Technologies Corporation), filtered (band-pass, 20 Hz to 2.5 kHz), and digitized at 5 kHz (Micro 1401 Cambridge Electronics Design). The peak-to-peak amplitude of the MEPs (mV) occurring between 15 and 100 ms after the TS were measured for each trial. Trials in which test pulses coincided with motor activity or failed to elicit reliable MEPs (i.e., value exceeded 1.5 times the interquartile range for the participant) were removed from the analysis (~2% of trials). The mean MEP amplitude for paired-pulse stimulation (CS-TS) was normalized by calculating the ratio of the amplitude relative to the mean single-pulse TS alone to M1 for each participant. Separate split-plot analysis of variances (ANOVAs) were carried out on the normalized MEP amplitudes for each PPC stimulus-response curve at each PPC stimulation intensity using Age (two levels: young or older adults) as a between-subjects factor and ISI (three levels: 4, 6, or 8 ms) as a within-subjects factor. The Bonferroni method was used for *post-hoc t*-test comparisons. The Greenhouse-Geiser method was used to correct for sphericity. Independent sample *t*-test was used to compare motor excitability and behavioral measures between groups. Paired *t*-tests also were conducted on the absolute amplitudes of the test MEP and conditioned MEP for the PPC stimulation intensity of 90% RMT at 6 ms ISI to evaluate facilitation and inhibition within the older and young adults. Correlations between neurophysiological and behavioral data were tested with Pearson's coefficient. Statistical analysis was performed using IBM-SPSS Statistics Version 26. A significance threshold was set at *p* < 0.05. Partial η squared ($\eta_{\text{p}}^{\;2}$) values were computed as a measure of effect size. Cutoffs for effect sizes are considered small (≥0.01), medium (≥0.06), and large (≥0.14) (Cohen, 1992). Mean and standard error values are reported.

## Results

As shown in Figure 1, no significant age difference was found in measures of motor excitability (all *t*_24_ < 0.71, all *p* > 0.49). The RMT of maximum stimulator output (MSO) was 45.2 ± 2.0% MSO for young adults and 44.8 ± 2.5% MSO for older adults (Figure 1B). The intensity to elicit a MEP amplitude of 1 mV in the right-hand target muscle was 48.3 ± 2.4% MSO for young adults and 48.4 ± 3.7% MSO for older adults (Figure 1C).

**Figure 2:**
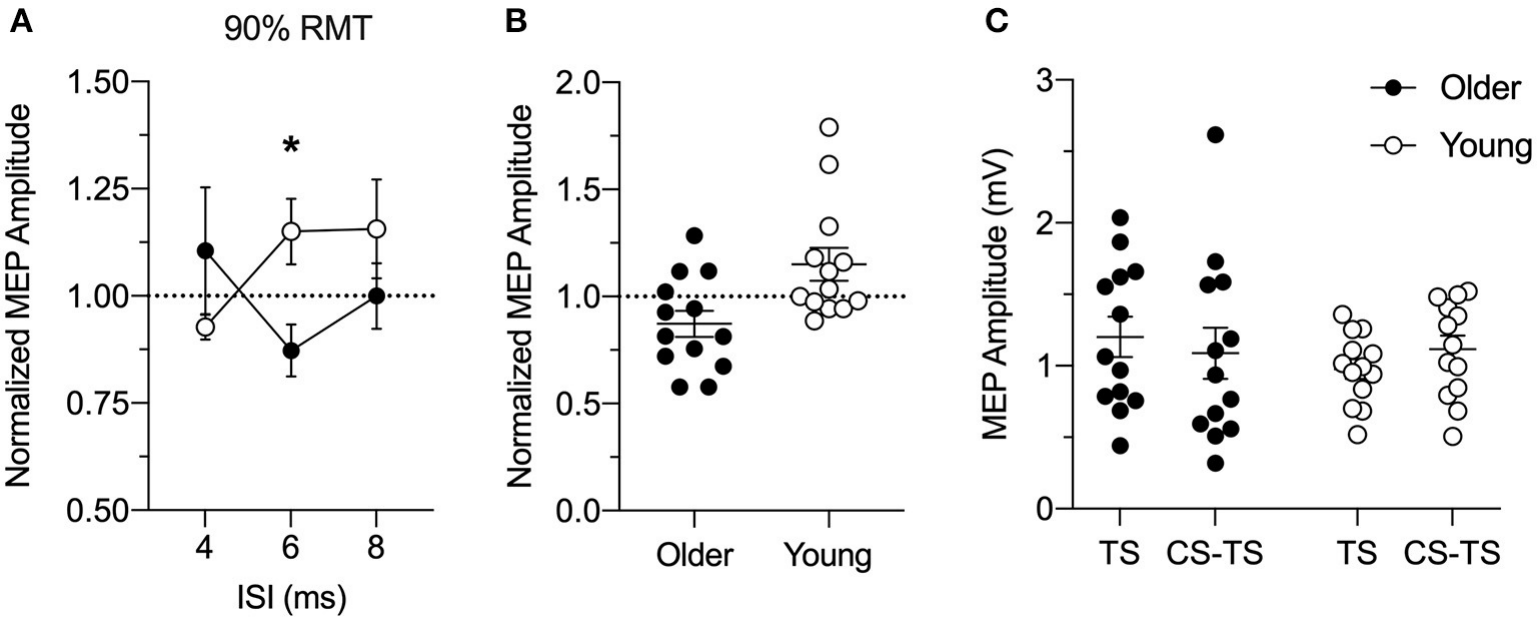
**(A)** Group analysis of the effects of left posterior parietal cortex (PPC) stimulation at 90% resting motor threshold (RMT) intensity on motor evoked potential (MEP) amplitude induced by left M1 stimulation for older (*n* = 13; closed circles) and young (*n* = 13; open circles) adults at rest. The conditioning stimulus (CS) to PPC preceded a test stimulus (TS) to the primary motor cortex (M1) by an inter-stimulus interval (ISI) of 4, 6, or 8 ms. MEP amplitude was normalized as a ratio of the MEP amplitude evoked by paired-pulse stimulation (CS-TS) to that evoked by single-pulse stimulation (TS) to M1 alone (dashed line). Y = 1 indicates no effect of TMS to PPC on M1 excitability, whereas ratios higher than 1 indicate increased and ratios lower than 1 indicate decreased M1 excitability because of PPC stimulation. Facilitation in the ratio of MEP amplitude in young adults was reduced in older adults with PPC stimulation delivered at an intensity of 90% RMT at ISI of 6 ms. **(B)** Individual conditioned MEP amplitudes for the stimulus-response curve at 90% of RMT at the 6 ms ISI for older (*n* = 13, filled circles) and young (*n* = 13, open circles) adults normalized to TS alone (dashed line). **(C)** Individual absolute values of MEP amplitudes (mV) for the single-pulse TS to M1 and paired-pulse CS-TS to PPC at an intensity of 90% RMT at the 6 ms ISI for older (filled circles) and young (open circles) adults. Mean and SE are presented. *p < 0.05.

Figure 2A shows PPC-M1 connectivity in young and older adults. Facilitation seen in the MEP amplitude ratio in young adults was reduced in older adults with PPC stimulation at 90% RMT for the ISI of 6 ms (significant Age and ISI interaction: *F*_(1.96,47.0)_ = 3.4, *p* = 0.043, $\eta_{\text{p}}^{\;2}$ = 0.12; no main effect of Age: *F*_(1,24)_1.17, *p* = 0.29, $\eta_{\text{p}}^{\;2}$ = 0.05; no main effect of ISI: *F*_(1.96,47.0)_ = 0.34, *p* = 0.71, $\eta_{\text{p}}^{\;2}$ = 0.01). Specifically, *post hoc* tests confirmed that the ratio of the MEP amplitude was significantly different between the age groups for the PPC stimulus-response curve at 90% RMT at the 6 ms ISI (Bonferroni's *t*-test: *t*_22.9_ = 2.84, *p* = 0.028). The results show that the facilitation between left PPC and ipsilateral M1 connections in young adults is reduced in older adults. Closer inspection of the individual normalized data confirmed that left PPC stimulation intensity of 90% RMT at 6 ms ISI caused inhibition of corticospinal excitability in ipsilateral M1 in about 69% of the older adults (Figure 2B). A similar pattern emerged at this timing and intensity when comparing the absolute amplitude of the conditioned and test MEP (Figure 2C). Paired *t*-tests revealed a trend toward significance in the facilitation of the conditioned MEP compared to TS alone in young adults (*t*_12_ = 2.04, *p* = 0.06), while the analysis within older adults did not reach significance (*t*_12_ = 1.38, *p* = 0.19). It is worth noting that single-pulse TS MEPs for the older adults were more variable than young adults, possibly influencing the normalized MEP amplitude ratio.

**Figure 3:**
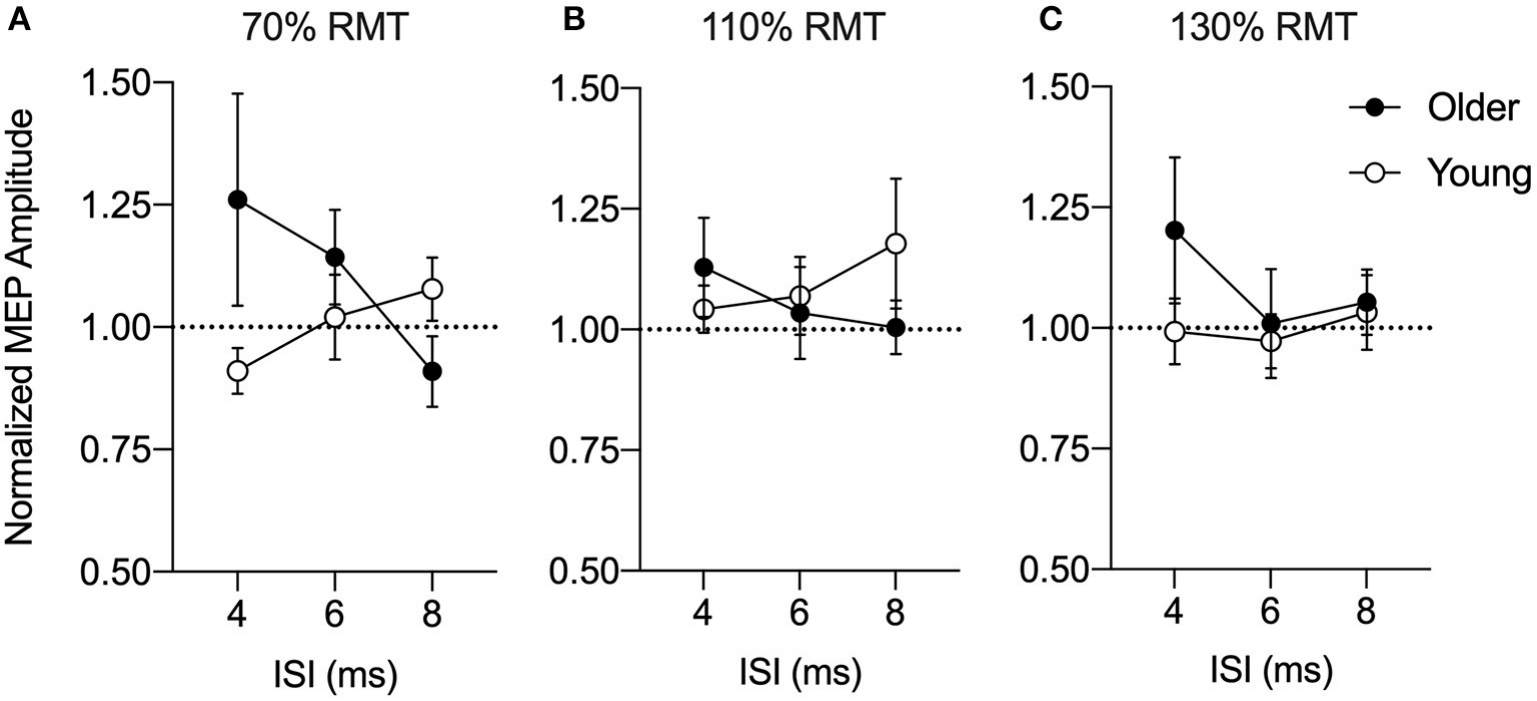
Group-averaged conditioned motor evoked potential (MEP) amplitudes for a posterior parietal cortex (PPC) stimulus-response curve at 70% **(A)**, 110% **(B)**, and 130% **(C)** of resting motor threshold (RMT) at an inter-stimulus interval (ISI) of 4, 6, or 8 ms normalized to a test stimulus (TS) alone (dashed line). MEP amplitudes are expressed as a ratio of the amplitude relative to the mean single-pulse TS alone to the primary motor cortex (M1) for older (*n* = 13, filled circles) and young adults (*n* = 13, open circles). Mean and SE are presented.

In a series of control experiments, we also verified whether the PPC-M1 connectivity at rest would differ with different PPC stimulation intensities. In each case, no significant age difference was found in the PPC stimulus-response curve at intensities of 70, 110, or 130% RMT (Figure 3). At 70% RMT (Figure 3A), a split-plot ANOVA showed that there was no significant effect of Age [*F*_(1,24)_ = 1.42, *p* = 0.25, $\eta_{\text{p}}^{\;2}$ = 0.06], ISI (*F*_(1.44,34.6)_ = 0.40, *p* = 0.60, $\eta_{\text{p}}^{\;2}$ = 0.02), nor an interaction effect [*F*_(1.44,34.6)_ = 2.52, *p* = 0.11, $\eta_{\text{p}}^{\;2}$ = 0.10]. At 110% RMT (Figure 3B), a split-plot ANOVA showed that there was no significant effect of Age [*F*_(1,24)_ = 0.28, *p* = 0.60, $\eta_{\text{p}}^{\;2}$ = 0.01], ISI [*F*_(1.73,41.5)_ = 0.11, *p* = 0.90, $\eta_{\text{p}}^{\;2}$ = 0.005], nor an interaction effect [*F*_(1.73,41.5)_ = 1.05, *p* = 0.35, $\eta_{\text{p}}^{\;2}$ = 0.04]. Similarly, at 130% RMT (Figure 3C), a split-plot ANOVA showed that there was no significant effect of Age [*F*_(1,24)_ = 1.32, *p* = 0.26, $\eta_{\text{p}}^{\;2}$ = 0.05], ISI [*F*_(1.93,46.29)_ = 0.64, *p* = 0.53, $\eta_{\text{p}}^{\;2}$ = 0.03], nor an interaction effect [*F*_(1.93,46.29)_ = 0.61, *p* = 0.54, $\eta_{\text{p}}^{\;2}$ = 0.03].

**Figure 4:**
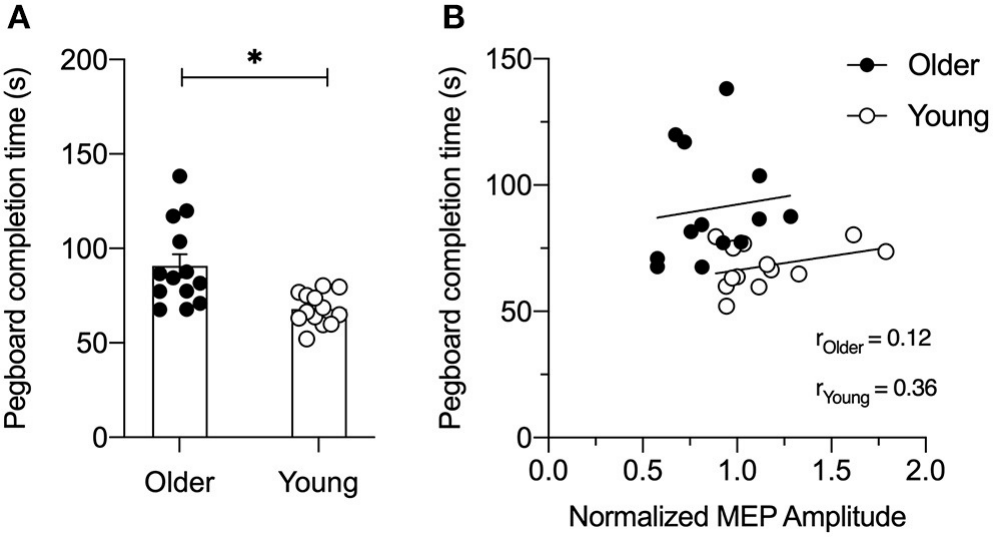
**(A)** Bar graph for group averaged completion times for the Grooved Pegboard Test in seconds (s). Individual data are presented for older (filled circles) and young (open circles) adults. As expected, young adults performed the task faster compared to older adults. Error bars represent SE. *p < 0.05. **(B)** Correlation between normalized motor evoked potential (MEP) at the 6 ms inter-stimulus interval (ISI) for PPC stimulus-response curve at 90% resting motor threshold (RMT) and Grooved Pegboard Test completion time for each older (filled circles) and young adult (open circles). A simple linear regression line is superimposed over the individual data points for each group. Normalized MEP amplitude did not correlate with the Grooved Pegboard Test performance in both young and older adults (*all p's* > 0.2).

As shown in Figure 4A, young adults were significantly faster than older adults at completing the GPT (young adults = 68.0 ± 8.6 s vs. older adults = 90.7 ± 22.2 s, *t*_24_ = 3.44, *p* = 0.003). Figure 4B shows associations between the PPC-M1 connectivity at rest and motor skill performance in young and older adults. A correlation analysis for each age group showed that the normalized MEP amplitude for the PPC stimulation intensity of 90% RMT at the 6 ms ISI did not correlate with GPT completion time in both young (*r* = 0.130; *p* = 0.23) and older adults (*r* = 0.015; *p* = 0.69).

## Discussion

A reduction of facilitation in a parieto-motor connection responsible for skilled hand movements was demonstrated in older adults compared to young adults. Using a dsTMS approach, we found that a conditioning stimulus to PPC with a 90% RMT intensity delivered 6 ms prior to a test stimulus to M1 resulted in a facilitation of the MEP amplitude in young but not older adults. In fact, the PPC-M1 interaction in the older group changed from facilitation to inhibition. The present findings are in line with prior dsTMS work in young adults showing short-latency PPC-M1 functional interactions are selectively facilitated at rest when applying CS to PPC at an intensity of 90% RMT (Koch et al., 2007; Karabanov et al., 2013). In the current study, the reduced PPC-M1 facilitation in older adults may be related to impairments of high-level movement planning signals in PPC as demonstrated by their slowed completion time of the GPT (Andersen and Cui, 2009). However, future work will need to better characterize the relationship between functional PPC-M1 connectivity and skilled motor performance. For instance, previous dsTMS studies have shown that functional PPC-M1 connectivity is modulated early in the motor plan for different types of hand actions at a similar ISI and conditioning stimulation intensity in young adults (Koch et al., 2008; Vesia et al., 2013, 2017). Yet, no work has investigated whether there is a relationship between functional PPC-M1 interactions during grasp preparation and manual dexterity in older adults. Future investigation is needed to determine whether similar age-related differences occur during these action-associated processes. It also should be noted that it remains unclear whether the non-significant association between reduced facilitation of parieto-motor functional connections and manual dexterity in older adults is present in a larger sample size given the moderate sample size in the current study. One possible explanation of the current results is that widespread motor plans involving multiple, parallel parieto-premotor-motor circuits could modulate corticospinal output associated with sensorimotor hand control (Koch and Rothwell, 2009; Davare et al., 2010, 2011; Vesia and Davare, 2011; Turella and Lingnau, 2014; Vesia et al., 2018). It also is possible that the relationship between cortico-cortical connections in the motor system and skilled hand behavior likely encompasses a much broader range of brain regions within frontal, parietal, and temporal cortices to support the flexibility of human hand behavior (Grafton, 2010; Gallivan and Culham, 2015; Monaco et al., 2020; Turella et al., 2020).

This interpretation is in line with prior dsTMS studies demonstrating age-related decline between M1 and frontal areas in the motor cortical network such as dorsolateral prefrontal cortex (Fujiyama et al., 2016), SMA (Green et al., 2018), and PMd (Ni et al., 2014). Together, these findings suggest an age-related reduction in functional connectivity from action-associated cortical areas to M1 in older adults. It is possible that the degraded facilitatory inputs from PPC to M1 in older adults likely represent an early marker of age-related decline for functional connectivity underlying complex motor skills. This view is in line with theoretical suggestions that older adults recruit frontal cortical areas to compensate for bottom-up sensory processing in posterior cortical areas when performing more cognitive-demanding motor tasks (Davis et al., 2008). Indeed, a large body of research provides complementary evidence linking these age-related differences in functional activation and connectivity patterns with cognitive and motor performance (see reviews Seidler et al., 2010; Damoiseaux, 2017). Functional neuroimaging studies have consistently demonstrated enhanced prefrontal influences on the motor system in response to increased task demands in older adults (Heuninckx et al., 2005; Wu and Hallett, 2005; Cabeza et al., 2018). One such study found that prefrontal areas compensated for decreased parietal influences on premotor areas associated with a decline in perceptual motor speed with advancing age (Michely et al., 2018). This is also consistent with evidence linking age-related decline in cognitively demanding motor tasks with structural changes in white matter tracts that connect sensorimotor, frontal, and parietal regions in older adults (Stewart et al., 2014). The reduced efficacy of this connection is also supported by findings in individuals with Parkinson's disease (Palomar et al., 2013) and stroke (Schulz et al., 2015) that show a more favorable motor outcome related to higher levels of communication between frontal and parietal areas in the motor system. Additionally, dsTMS evidence has shown that this selective age-related decrease in PPC-M1 facilitation in healthy older adults is exacerbated at early clinical stages of Alzheimer's disease (Bonnì et al., 2012). This decreased functional connectivity precedes disease-related changes in cognitive-related frontal areas and could in part represent a key driver of cognitive decline in Alzheimer's disease (Koch et al., 2019).

It is worth noting that neurobiological aging is a complex process involving interactions between local cortical and brain network plasticity (Freitas et al., 2013). In the case of aging, compensatory processes likely depend on the neural reorganization and recruitment of alternative circuits to attenuate cognitive and motor declines that occur with age (Cabeza et al., 2018). Future longitudinal studies delineating the specific contribution of PPC on motor related frontal circuits combining TMS with neuroimaging approaches will be needed to gain insight into the mechanisms of system-level plasticity across the lifespan. We recognize that age-related neural decline such as brain atrophy, synaptic loss, and white matter degradation could account for the observed MEP amplitude differences between young and older adults (Giorgio et al., 2010; Farokhian et al., 2017). However, we believe a global effect on mechanisms underlying aging is unlikely to account for the highly specific reduction in PPC-M1 facilitation in the current study. A methodological limitation of the study is that we did not selectively localize PPC sites for hand actions at the individual level using fMRI or task-based dsTMS. Prior dsTMS work has shown anatomical and functional differences in connectivity between PPC regions and M1 (Karabanov et al., 2013). This raises the possibility that the effects at other ISIs and conditioning stimulation intensities in our study could be dependent on different neural substrates. Further studies will need to examine the effect of nearby parietal regions on motor excitability to account for the individual differences in functional connectivity among older adults. Finally, we recognize that the older adults tested in the current study were high-functioning and in relatively good health based on self-reports. Therefore, future work is needed with a more heterogenous subset of older participants to clarify the effects of physical activity and other environmental factors on age-related cognitive and motor deficits.

We conclude that the reduced PPC-M1 facilitation in older adults may be an early marker of age-related decline in the neural control of movement. Our findings could have implications for understanding functional parieto-frontal connectivity affected by advancing age in both healthy and clinical populations. Importantly, dsTMS methods could be used to develop better diagnostic tools and treatment approaches (Fox et al., 2012, Hallett et al., 2017; Goldenkoff et al., 2020). We propose that prospective strengthening of PPC-M1 circuitry in healthy adults might be a fruitful therapeutic path to counteract the gradual age-related breakdown in functional connectivity within the motor-related network associated with motor impairments. It is possible that the preservation of these neural substrates could enhance resilience of the intact circuitry and minimize compensatory shifts in brain networks that maintain optimal cognitive and motor performance.

## Data Availability Statement

The raw data supporting the conclusions of this article will be made available by the authors, without undue reservation.

## Ethics Statement

The studies involving human participants were reviewed and approved by University of Michigan Institutional Review Board. The patients/participants provided their written informed consent to participate in this study.

## Author Contributions

EG: methodology, formal analysis, investigation, writing—original draft, writing—review & editing, visualization, and project administration. RL: methodology, investigation, project administration, formal analysis, and writing—review & editing. SB: methodology, resources, writing—review & editing, and supervision. MV: conceptualization, methodology, resources, writing—review & editing, supervision, and funding acquisition. All authors contributed to the article and approved the submitted version.

## Conflict of Interest

The authors declare that the research was conducted in the absence of any commercial or financial relationships that could be construed as a potential conflict of interest.
